# Association between systemic immune-inflammation index and diabetes: a population-based study from the NHANES

**DOI:** 10.3389/fendo.2023.1245199

**Published:** 2023-10-31

**Authors:** Yiqi Nie, Haiting Zhou, Jing Wang, Hongxing Kan

**Affiliations:** ^1^ School of Medical Information Engineering, Anhui University of Traditional Chinese Medicine, Hefei, Anhui, China; ^2^ Anhui Computer Application Research Institute of Chinese Medicine, China Academy of Chinese Medical Sciences, Hefei, Anhui, China; ^3^ School of Chinese Medicine, Anhui University of Traditional Chinese Medicine, Hefei, Anhui, China

**Keywords:** systemic immune-inflammation index, diabetes, NHANES, cross-sectional study, population-based study

## Abstract

**Background:**

Systemic Immune-Inflammation Index (SII) has been reported to be associated with diabetes. We aimed to assess possible links between SII and diabetes.

**Methods:**

Data were obtained from the 2017-2020 National Health and Nutrition Examination Survey (NHANES) database. After removing missing data for SII and diabetes, we examined patients older than 20 years. Simultaneously, the relationship between SII and diabetes was examined using weighted multivariate regression analysis, subgroup analysis, and smooth curve fitting.

**Results:**

There were 7877 subjects in this study, the average SII was 524.91 ± 358.90, and the prevalence of diabetes was 16.07%. Weighted multivariate regression analysis found that SII was positively associated with diabetes, and in model 3, this positive association remained stable (OR = 1.04; 95% CI: 1.02–1.06; p = 0.0006), indicating that each additional unit of SII, the possibility of having diabetes increased by 4%. Gender, age, BMI, regular exercise, high blood pressure, and smoking did not significantly affect this positive link, according to the interaction test (p for trend>0.05).

**Discussion:**

Additional prospective studies are required to examine the precise connection between higher SII levels and diabetes, which may be associated with higher SII levels.

## 1 Introduction

The chronic metabolic disorder known as diabetes is characterized by persistently high blood sugar levels (1). Worldwide, the prevalence of diabetes is rising each year and has emerged as a severe public health issue (2). More than 460 million people worldwide have diabetes, and type 2 diabetes affects more than 90% of these individuals. Diabetes harms patients’ health but also has a significant financial impact on families and society (3). Diabetic microangiopathy, one of the most prevalent early consequences of diabetes, is also a risk factor for heart and renal disease (4). Additionally, the study finds levels of systemic inflammation may explain the increased prevalence of diabetes (5).

Systemic Immunity-Inflammation Index (SII) is the platelet count multiplied by the neutrophil count divided by the lymphocyte count, which is considered to be a new and reliable indicator (6) for comprehensively measuring the systemic immunity and inflammation level of the subject (7). Inflammatory factor indicators have been linked to diabetes, according to an increasing number of research (8). Jie Wang et al. investigated the relationship between NLR and depression in patients with diabetes and found that NLR increased and the prevalence of depression increased (9). Bartosz Hudzik et al. find a link between PLR and diabetes (10). In diabetic patients, Dan Yu et al. discovered that LMR can be used as an index to predict the recurrence of rectal cancer (11). These indicators, which only represent two different types of immune cells, could not be very predictive. According to Jie Wang et al., SII is a risk factor for diabetes depression (12). It can be assumed that there may be a relationship between SII and diabetes since SII has been suggested as an index to detect depression in diabetes. However, no one has independently looked into the connection between SII and diabetes.

Liu Yongming et al. found that middle-aged and elderly diabetes caused by arteriosclerosis may be mediated by white blood cell count (13). Xiang Fang et al. Diabetes is related to the expression of inflammatory genes and white blood cell count, and blood glucose is positively correlated with inflammatory genes and white blood cell count (14). Francesco Zaccardi et al. found that compared with patients without diabetes, there was no difference in platelet count in patients with diabetes. However, this study still took into account the image of platelet count in the experiment and included it in the covariate to design the experiment (15).

The link between SII and diabetes will be examined in this article. We examine the association between SII and diabetes using data from the NHANES database for the years 2017 to 2020. We think that there may be a connection between the rise in SII and the rise in diabetes prevalence. Researchers and medical professionals offer references.

## 2 Materials and methods

### 2.1 Population research

**Figure 1:**
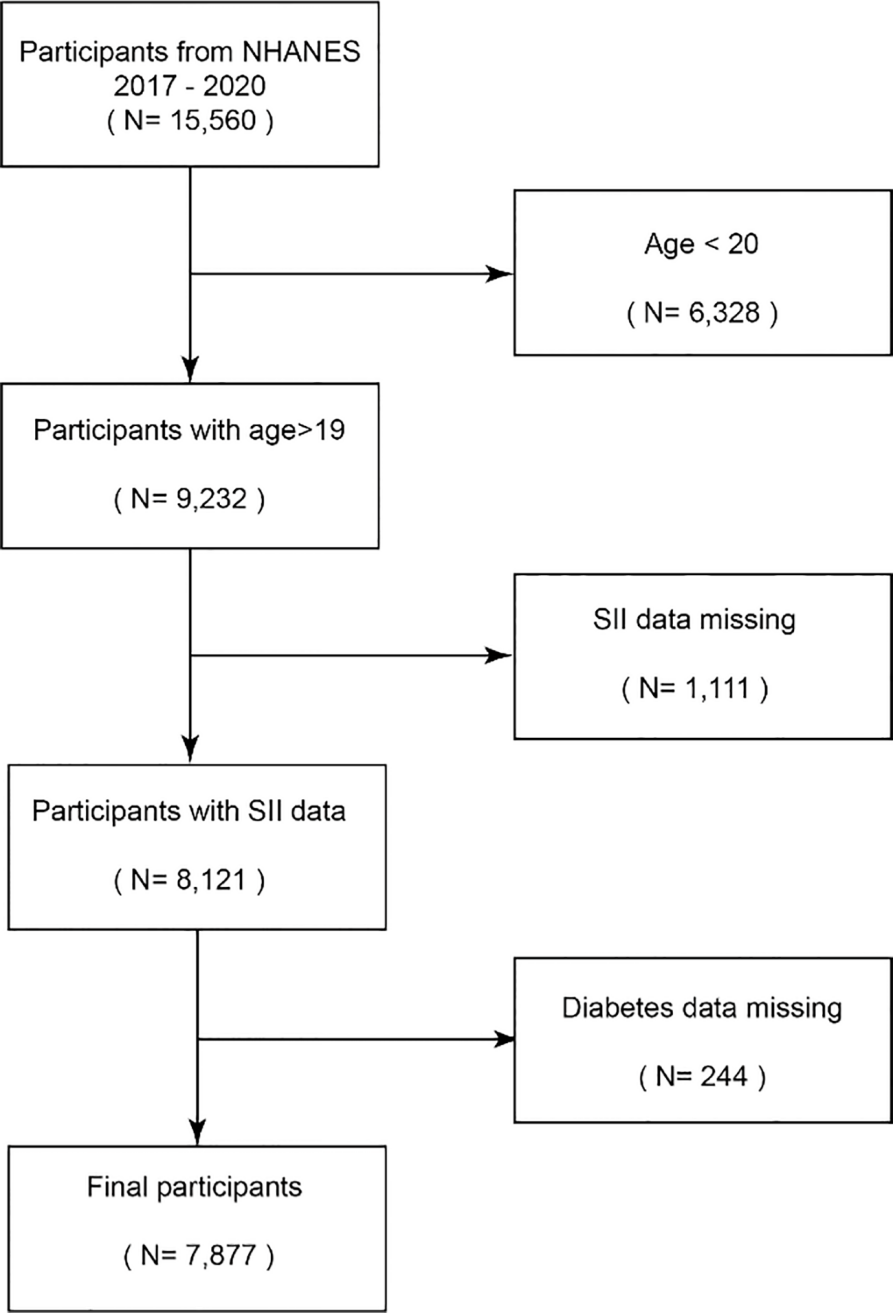
Flowchart of the sample selection from NHANES 2017–2020.

The National Health and Nutrition Examination Survey (NHANES) is a research program designed to assess adults’ and children’s health and nutritional status in the United States (16). The NHANES database contains population data, questionnaire data, laboratory data, and dietary data. This study is used to determine the prevalence and pathogenic factors of diseases, etc., and provides national standard references to help design sound public health policies (17). In total, 15,560 participants from the 2017–2018 and 2019–2020 research years were included in this study, whereas 6,328 participants under the age of 20 were omitted. Following the exclusion of 1111 people with missing SII data and 244 participants with missing diabetes data, 7877 participants were ultimately included in the study. Subjects were given the go-ahead to participate in the NHANES by the NCHS Ethics Review Board, and each participant gave their written informed consent (18) (**Figure 1**).

### 2.2 Exposure variables and outcome variables

SII is a composite index made up of platelet count, neutrophil count, and lymphocyte count that is used as an exposure variable and is assessed using an automated hematology analysis instrument (CoulterDxH 800 analyzer). calculated by dividing the lymphocyte count by the platelet count, and then multiplying by the neutrophil count (19).

Did your doctor inform you that you had diabetes? Are these other questions asked by a professional interviewer utilizing a computer-assisted personal interview (CAPI) system at home? The subject was deemed to have diabetes if his response was yes. Having diabetes diagnosed by a doctor was intended to be an outcome variable (20).

### 2.3 Covariates

We summarized potential covariates that might confound the association between SII and diabetes. The covariates selected for this study were: age(year), Albumin refrigerated serum, White blood cell count, Platelet count, blood urea nitrogen content, chloride content, dietary protein intake, carbohydrate intake, total sugar intake, total fat intake, cholesterol intake, gender, race, education level (divided into no high school education, high school education, high school education or above), marital status (married with a partner, divorced and separated, widowed, never married), poverty-income ratio (0-1.5, 1.5-3.5, >3.5), BMI (0-25, 25-30, >30), whether you have high blood pressure, whether you smoke (21), whether you exercise regularly (22). Among them, the BMI classification corresponds to three groups of normal weight, overweight, and obese. Refrigerated serum albumin is measured in g/L, and albumin concentration is measured using the dye bromocreol violet (BCP). The PH value binds to albumin in the PH range of 5.2-6.8, and the color change is measured at 600nm, with secondary measurements at 700nm. Albumin value measurement is used in the diagnosis and treatment of certain liver and heart diseases as well as diabetes (23). Both the White blood cell count (24) and the Platelet count (25) are measured in 1000 cells/uL. A complete blood count on a blood specimen is measured at a mobile testing center using a Beckman Coulter DxH 800 instrument. The VCS (volume, conductivity, and dispersion) method was used to determine the leucocytes. Blood urea nitrogen was measured at 340nm using a coupled enzyme reaction in the unit of mmol/L (26). The chloride content was measured in mmol/L, and the serum electrolyte concentration was determined by the indirect ion selective electrode (ISE) method. Sample dilution 1:31 (27). Dietary protein intake, carbohydrate intake, total sugar intake, total fat intake, and cholesterol intake, these data are from the dietary section of the database. Dietary recall interviews were collected at a mobile test center (MEC), and participants’ total nutrient intake on day one was used in this study (28). High blood pressure was determined by the source of the questionnaire part, whether or not the doctor considered having high blood pressure (29). Whether you smoke or not, according to the part of the questionnaire, whether you have smoked 100 cigarettes so far (30).

### 2.4 Statistical methods

Continuous variables were expressed as means with standard deviations in the description of the study population, while categorical variables were expressed as percentages. The association between participants in the SII/100 quartile group and the presence of diabetes was assessed using the t-test for continuous variables and the chi-square test for categorical variables. To investigate the relationship between SII and the prevalence of diabetes, multivariate logistic regression was performed. Model 1 had no covariates, model 2 added covariates age, sex, and race, and model 3 adjusted variables: age, sex, race, urine albumin content (31), refrigerated serum content, blood urea nitrogen content (32), chloride content, dietary protein intake, carbohydrate intake, total sugar intake, total fat intake, cholesterol intake, education Degree (divided into no high school education, high school education, high school education or above), marital status (married with a partner, divorced and separated, widowed, never married), poverty-income ratio (0-1.5, 1.5-3.5, >3.5), BMI (0-25, 25-30, >30), whether you have high blood pressure (33), whether you smoke, whether you exercise regularly. SII and diabetes were assessed in the model using odds ratios (ORs) and 95% confidence intervals (CIs). Create a multivariate test by controlling variables and fitting smooth curves to three models. The relationship and inflection point between SII and diabetes were examined using a threshold effect analysis model. R Studio (version 4.2.2) and empowered stats (version 2.0) were used to do the statistical study. 0.05 was the threshold for significance. We employ a weighting approach to lower the dataset’s volatility.

## 3 Results

### 3.1 Baseline characteristics of the study population

A total of 7877 subjects were included in this study, of which 48.14% were male and 51.86% were female, with an average age of 50.87 ± 17.56. The mean value of SII was 524.91 ± 358.90, and the prevalence of diabetes was 16.07%.

**Table 1** is a table of diabetes-based weighted demographic baseline characteristics. The average age of subjects with diabetes was 61.09 ± 13.16, and the average age of subjects without diabetes was 46.55 ± 17.15. The average age of subjects with diabetes was higher than that of subjects without diabetes. Diabetes is used as a stratified variable. The presence or absence of diabetes is related to age, gender, education level, marital status, poverty-income ratio, BMI, hypertension, smoking or not, regular exercise, SII, albumin content, blood urea nitrogen content, Chloride content, dietary carbohydrate intake, dietary total sugar intake, and dietary cholesterol intake were significantly correlated (p<0.05). Compared with people without diabetes, people with diabetes tended to be older, high school educated, married or in a partner, had higher SII, higher albumin, higher blood urea nitrogen, lower chloride, carbohydrate non-Hispanic white males with lower intake, lower sugar intake, higher cholesterol intake, poverty-income ratio >3.5, BMI >30 kg/m2, hypertension, smoking, and no regular exercise. People with diabetes have higher white blood cell counts and lower platelet counts than those without diabetes.

**Table 1:** Baseline characteristics of study population according to Diabetes, weighted.

Characteristics	Diabetes	Non-Diabetes	*p*-Value
	N=1266	N=6611	
Age(years)	61.09 ± 13.16	46.55 ± 17.15	<0.0001
SII	597.58 ± 419.76	532.32 ± 334.90	<0.0001
White blood cell count (1000 cells/uL)	7.61 ± 2.24	7.17 ± 5.38	0.004
Platelet count (1000 cells/uL)	239.47 ± 71.08	247.11 ± 64.54	<0.001
Albumin, refrigerated serum (g/L)	39.71 ± 3.52	41.13 ± 3.33	<0.0001
Blood Urea Nitrogen (mmol/L)	6.40 ± 2.78	5.18 ± 1.80	<0.0001
Chloride (mmol/L)	100.43 ± 3.44	101.36 ± 2.64	<0.0001
Protein (gm)	79.53 ± 38.53	82.07 ± 42.82	0.0989
Carbohydrate (gm)	225.10 ± 107.48	249.89 ± 128.06	<0.0001
Total sugars (gm)	94.82 ± 70.74	108.12 ± 78.70	<0.0001
Total fat (gm)	87.98 ± 46.28	89.88 ± 49.46	0.2875
Cholesterol (mg)	341.46 ± 269.35	317.66 ± 254.35	0.0108
Gender (%)			<0.0001
Male	54.99	46.95	
Female	45.01	53.05	
Race/Ethnicity (%)			0.1288
Mexican American	9.29	8.36	
Non-Hispanic White	61.00	63.53	
Non-Hispanic Black	12.02	10.80	
Other Hispanic	6.50	7.80	
Other Race	11.20	9.50	
Education (%)			<0.0001
Less than high school	15.85	10.30	
High school	32.29	26.13	
More than high school	51.86	63.57	
Marital status (%)			<0.0001
Married/Living with partner	66.34	61.43	
Widowed/Divorced/Separated	25.90	17.66	
Never married	7.76	20.91	
Income to poverty ratio (%)			<0.0001
0–1.5	24.69	22.14	
1.5–3.5	27.08	32.94	
>3.5	62.77	38.99	
BMI (kg/m^2^) (%)			<0.0001
0–25	10.14	28.08	
25–30	27.08	32.94	
>30	62.77	38.99	
High blood pressure (%)			<0.0001
Yes	68.85	27.14	
No	31.15	72.86	
Smoke (%)			<0.0001
Yes	51.14	41.60	
No	48.86	58.40	
Vigorous work activity (%)			<0.0001
Yes	20.53	28.05	
No	79.47	71.95	

Mean ± SD for continuous variables: the p-value was calculated by the weighted linear regression model. % for categorical variables: the p-value was calculated by a weighted chi-square test. BMI, body mass index; SII, SII, Systemic Immune-Inflammation Index.

**Table 2** is a table of SII-based weighted demographic baseline characteristics. SII quartile subjects have significant differences in age, albumin content, chloride content, protein content, gender, race, education level, income-poverty ratio, BMI, hypertension, diabetes, etc. (p<0.05). Subjects in the fourth quartile of the SII quartile tend to be older, have lower protein intake, lower carbohydrate intake, high school education or higher, poverty-income ratio>3.5, BMI>30 kg/m2, have Hypertensive, non-Hispanic white females without diabetes. SII was also significantly different in the quartile group from white blood cell count and platelet count, with the fourth quartile having the highest count.

**Table 2:** Baseline characteristics of study population according to Systemic Immune-Inflammation Index quartiles, weighted.

Characteristics	SII Quartiles				*p*-Value
	Q1	Q2	Q3	Q4	
	N=1969	N=1969	N=1969	N=1970	
Age (years)	47.44 ± 17.03	46.97 ± 17.08	48.68 ± 17.60	49.91 ± 17.57	<0.0001
White blood cell count (1000 cells/uL)	6.27 ± 9.25	6.72 ± 1.71	7.41 ± 1.82	8.58 ± 2.37	<0.001
Platelet count (1000 cells/uL)	206.44 ± 51.65	234.37 ± 50.68	254.93 ± 56.85	287.78 ± 72.62	<0.001
Albumin, refrigerated serum (g/L)	41.46 ± 3.30	41.38 ± 3.07	40.87 ± 3.18	40.21 ± 3.78	<0.0001
Blood Urea Nitrogen (mmol/L)	5.34 ± 1.92	5.25 ± 1.68	5.39 ± 2.02	5.34 ± 2.25	0.1865
Chloride (mmol/L)	101.44 ± 2.64	101.26 ± 2.63	101.38 ± 2.69	100.94 ± 3.03	<0.0001
Protein (gm)	83.02 ± 41.49	84.82 ± 45.16	82.11 ± 41.00	77.36 ± 41.08	<0.0001
Carbohydrate (gm)	250.17 ± 123.91	246.80 ± 120.98	247.20 ± 131.08	244.14 ± 127.46	0.5832
Total sugars (gm)	106.06 ± 76.30	103.07 ± 70.45	107.94 ± 83.80	108.95 ± 79.97	0.1046
Total fat (gm)	90.00 ± 48.35	91.36 ± 50.80	89.34 ± 47.47	87.99 ± 49.52	0.2070
Cholesterol (mg)	325.85 ± 260.72	324.46 ± 257.25	320.42 ± 256.25	312.20 ± 251.48	0.3786
Gender (%)					<0.0001
Male	56.33	51.01	45.06	40.99	
Female	43.67	48.99	54.94	59.01	
Race/Ethnicity (%)					<0.0001
Mexican American	8.12	9.84	8.50	7.41	
Non-Hispanic White	52.65	62.78	66.09	69.31	
Non-Hispanic Black	19.97	10.02	8.76	6.78	
Other Hispanic	7.16	8.33	7.11	7.90	
Other Race	12.10	9.02	9.54	8.60	
Education (%)					0.0438
Less than high school	10.27	11.34	11.40	10.73	
High school	26.69	25.60	27.41	27.71	
More than high school	63.04	63.06	61.19	61.56	
Marital status (%)					0.0974
Married/Living with partner	60.88	63.15	63.31	60.56	
Widowed/Divorced/Separated	18.42	16.56	18.90	20.61	
Never married	20.69	20.29	17.79	18.83	
Income to poverty ratio (%)					0.0002
0–1.5	23.87	21.76	20.49	23.94	
1.5–3.5	29.09	29.59	31.91	34.06	
>3.5	47.04	48.65	47.60	41.99	
BMI (kg/m^2^) (%)					<0.0001
0–25	32.67	26.26	22.68	23.47	
25–30	34.15	34.41	30.86	29.98	
>30	33.18	39.33	46.45	46.55	
High blood pressure (%)					<0.0001
Yes	30.16	28.73	31.25	37.97	
No	69.84	71.27	68.75	62.03	
Smoke					0.0894
Yes	41.81	40.40	43.60	44.92	
No	58.19	59.60	56.40	55.08	
Vigorous work activity (%)					0.4710
Yes	28.00	27.35	28.30	25.13	
No	72.00	72.65	71.70	74.86	
Diabetes (%)					<0.0001
Yes	10.13	11.69	11.06	14.84	
No	89.87	88.31	88.94	85.16	

Mean ± SD for continuous variables: the p-value was calculated by a weighted linear regression model. % for categorical variables: the p-value was calculated by a weighted chi-square test. Q, quartile; BMI, body mass index; SII, Systemic Immune-Inflammation Index.

### 3.2 Relationship between SII and diabetes

Since the effect size was not obvious, we magnified the value of SII by 100 times to compare the relationship between SII/100 and diabetes. **Table 3** shows the multivariate regression analysis between SII/100 and diabetes. For diabetes, a positive association between SII/100 and diabetes was observed. In model 3, this positive association remained stable (OR = 1.04; 95% CI: 1.02–1.06; p = 0.0006), indicating that each unit increase in SII/100 increased the likelihood of having diabetes by 4%. In a sensitivity analysis, a fully adjusted model for the SII/100 quartile (OR = 1.31; 95% CI: 1.05-1.63; p = 0.0187) indicated a stable relationship between elevated SII and increased odds of developing diabetes. Compared with the first quartile, participants in quartile 4 had a 31% increased risk of developing diabetes. At the same time, the p for trend of the three models were all <0.05, which was statistically significant.

**Table 3:** Association between systemic immune-Inflammation index and diabetes.

	Crude Model (Model 1)	Partially Adjusted Model (Model 2)	Fully Adjusted Model (Model 3)
	OR (95% CI) *p*-Value	OR (95% CI) *p*-Value	OR (95% CI) *p*-Value
SII/100	1.04 (1.02, 1.05) ***	1.04 (1.02, 1.05) ***	1.04 (1.02, 1.06) ***
SII/100 quartiles			
Quartiles 1	Reference	Reference	Reference
Quartiles 2	1.06 (0.89, 1.27)	1.17 (0.97, 1.41)	1.10 (0.88, 1.37)
Quartiles 3	1.09 (0.92, 1.30)	1.21 (1.00, 1.46) *	1.10 (0.88, 1.37)
Quartiles 4	1.40 (1.19, 1.66) ***	1.59 (1.33, 1.91) ***	1.31 (1.05, 1.63) *
p for trend	<0.0001	<0.0001	0.0187

Model 1, no covariates were adjusted. Model 2, age, sex, and race were adjusted. Model 3, age, sex, race, marital status, income to poverty ratio, education level, drink, smoke, BMI, high blood pressure, blood urea nitrogen, White blood cell count, Platelet count, chloride, protein, carbohydrate, total sugars, total fat, cholesterol, and vigorous work activity were adjusted. 95% CI, 95% confidence interval; OR, odds ratio; SII, Systemic Immune-Inflammation Index. * p < 0.05, ** p < 0.01, *** p < 0.001; a p < 0.05 was considered statistically significant.

**Figure 2:**
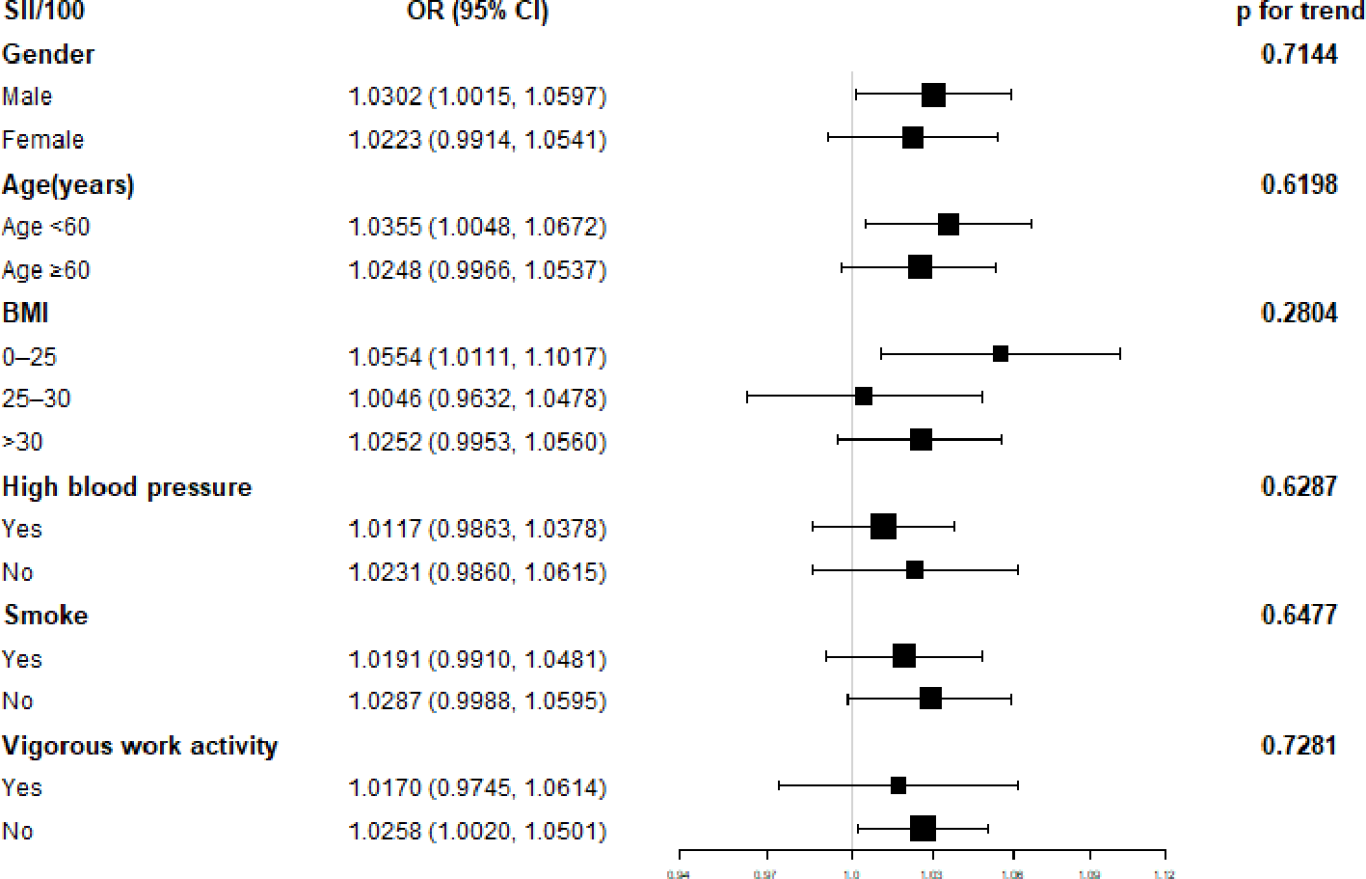
Subgroup analysis for the association between Systemic Immune-Inflammation Index and Diabetes.

A subgroup analysis of the association between SII and diabetes is shown in **Figure 2**. SII was significantly correlated with male sex, age less than 60, 0<BMI<25, and irregular exercise (p<0.05). The interaction test showed that there was no statistical difference in the relationship between SII and diabetes in each category, and gender, age, BMI, regular exercise, hypertension, and smoking had no significant impact on this positive relationship (p for trend>0.05).

**Figure 3:**
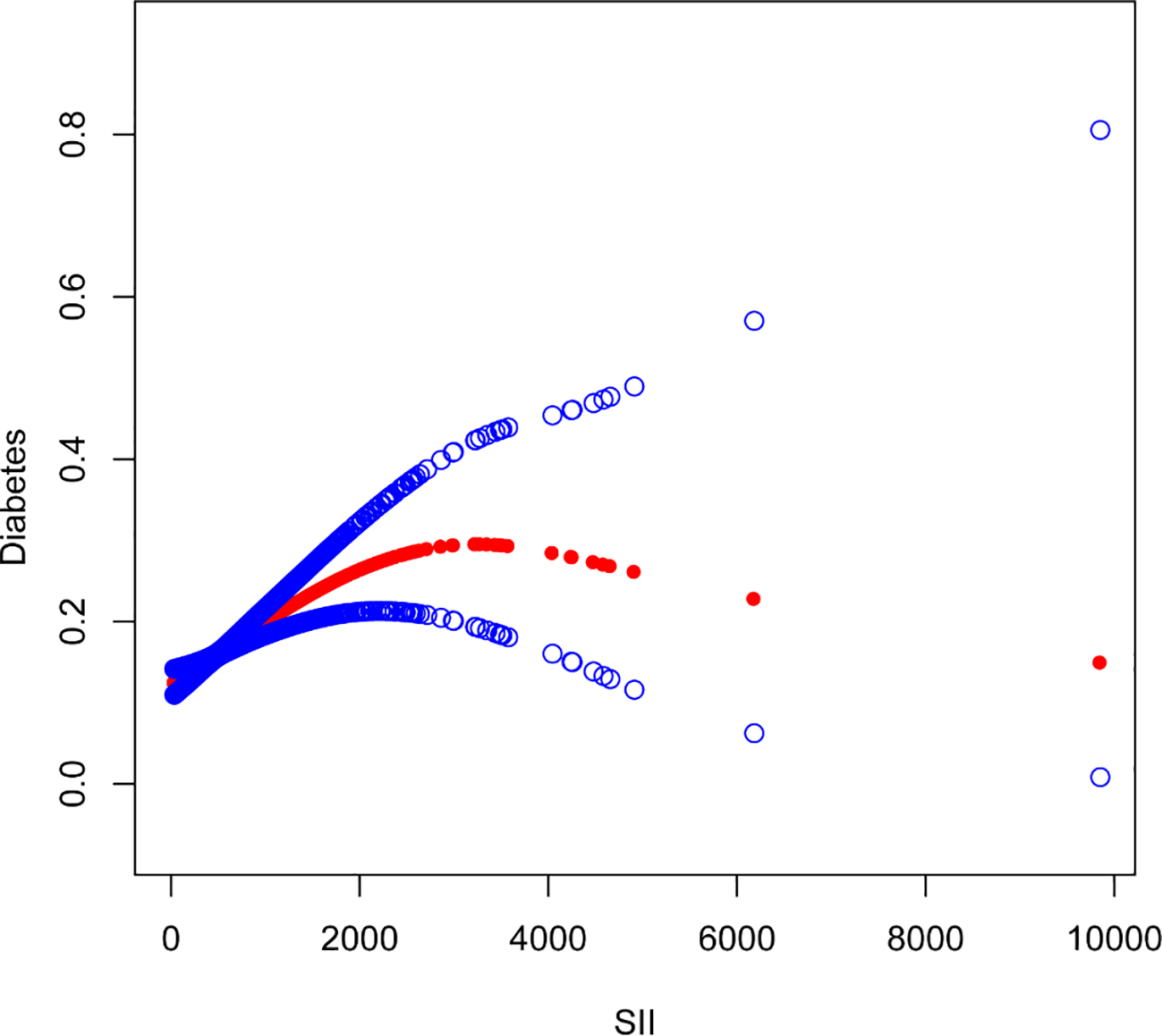
The association between Systemic Immune-Inflammation Index and Diabetes. The solid red line represents the smooth curve fit between variables. Blue bands represent the 95% confidence interval from the fit.

**Figure 4:**
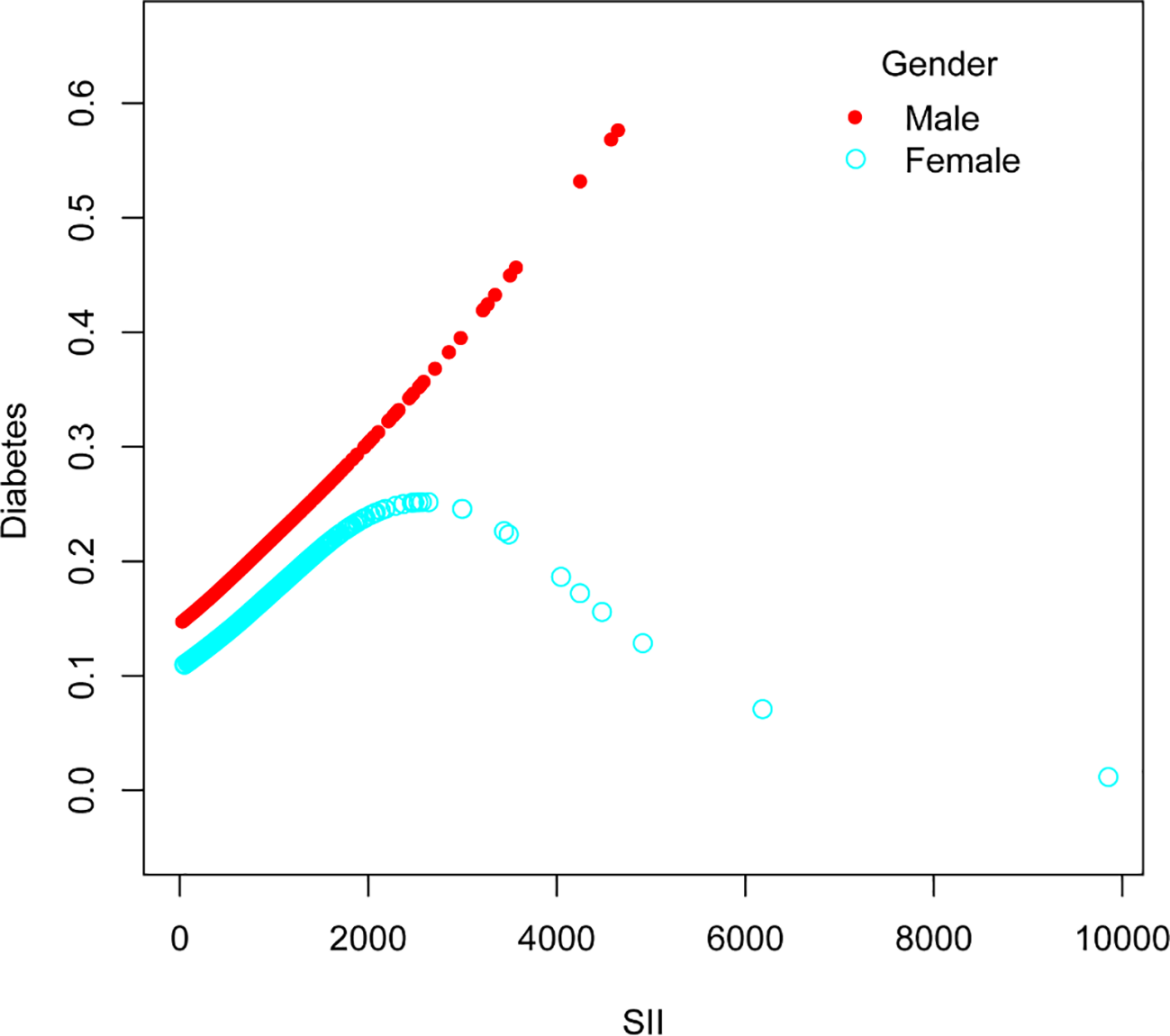
The association between Systemic Immune-Inflammation Index and Diabetes stratified by sex.

The nonlinear association between SII and diabetes was then described using smooth curve fitting (**Figures 3**, **4**). Adjusted variables: age, sex, education, marital status, poverty-income ratio, BMI, hypertension, smoking, regular exercise, albumin level, blood urea nitrogen level, chloride level, dietary carbohydrate intake, dietary total sugar intake, and dietary cholesterol intake, and high blood pressure. Interaction test showed that there was no statistically significant difference in the association between SII and diabetes among different stratification groups, and all interactions were p>0.05, indicating that age, sex, BMI, sampling, regular exercise, and hypertension had no significant dependence on this positive association. After stratified analysis by sex, it was found that the female stratification presents an inverted U-shaped curve.

## 4 Discussion

Our research revealed an association between increased SII and a higher prevalence of diabetes. After stratified analysis by gender, it was discovered that female stratification likewise exhibits an inverted U-shaped curve in the connection between SII and diabetes.

To our knowledge, few studies have individually assessed the association between SII and diabetes. Ahmet Elbeyli et al. found that SII can be used as a diagnostic marker for diabetic macular edema and improve diabetic retinopathy (34). Jie Wang et al. found that SII can be used as a diagnostic marker for diabetic depression (12). Wencong Guo et al. found that higher SII levels were significantly associated with diabetic nephropathy (35). Kübra Özata Gündoğdu et al. studied the association of diabetic macular edema with serous macular detachment and SII, and elevated SII levels may increase the incidence of serous macular detachment (36). Safak Ozer Balin et al. reported that SII can be used as a predictive marker for diabetic foot osteomyelitis (37). Yohanes Andy Rias et al. surveyed 294 Indonesian diabetic patients and found that low levels of SII can regulate psychological problems in diabetic patients (38). In contrast, Yohanes Andy Rias et al.’s research results showed a benign effect of low-level SII, which is different from the general high-level SII. Therefore, it is found that the level of SII may have different effects on the judgment of diabetes, and it is meaningful for us to study the relationship between SII and diabetes. Research by Jingxin Zhou et al. suggested that SII may be a potential marker for the treatment of diabetic macular edema (39). We hypothesize that there may be a relationship between SII and diabetes, which may be a good prospective marker, in light of the studies’ confirmation of SII’s predictive power. We discovered through our research that there is currently no research on the relationship between SII and diabetes alone, but there is research on the relationship between SII and diabetes-related diseases like the aforementioned diabetic macular edema, diabetic nephropathy, and diabetic depression. The studies mentioned above utilized several survey techniques and study populations at the same time. Our investigation discovered a potential link between greater SII levels and a higher chance of developing diabetes, which is consistent with the majority of studies.

Despite being a novel inflammatory marker, diabetes has not been examined with SII alone. However, diabetes has been linked in clinical studies with several traditional inflammatory indicators. Zhao-tong Jia et al. measured serum IMA and hs-CRP concentrations in patients with diabetic retinopathy by the rate-nephelometric method. There may be a positive correlation between hs-CRP concentration and the prevalence of diabetic retinopathy (r = 0.617, P < 0.01) (40). Hai-hang Liu et al. believed that serum hs-CRP concentration can predict the incidence of diabetes, and inflammatory factors play an important role in diabetes research (41). Klisic et al. studied the association of type 2 diabetes with some inflammatory factors, platelet-to-neutrophil ratio (PNR), monocyte/granulocyte-to-lymphocyte ratio (M/GLR), derived neutrophils to lymphocytes Cell ratio (dNLR), all three indicators are independently associated with type 2 diabetes (42). Klisic’s study affirmed the role of traditional inflammatory factors in the prediction of diabetes but did not specifically explore the correlation between the two, so in-depth research in this study is necessary. Si-Yang Wang et al. demonstrated that the ratio of neutrophils to lymphocytes can be used as a marker for predicting diabetes (43). Si-Yang Wang’s research also affirmed the outstanding work of inflammatory factors in the diagnosis of diabetes but did not conduct specific correlation studies. Mohamad Akbari’s group (44) and Dan Qu’s group (45) both considered elevated levels of interleukin 6 (IL-6) to be an independent predictor of diabetes. Compared with traditional inflammatory factors, SII binds three types of immune cells, reflects the inflammatory state well comprehensively, and has shown better prognostic value in several studies (46). For example, Afiat Berbudi et al. studied the effect of SII, neutrophil/lymphocyte ratio (NLR), platelet/lymphocyte ratio (PLR), and monocyte/lymphocyte ratio (MLR) in predicting the impact of type 2 diabetes on the immune system. Their ROC curve analysis confirmed that among these markers, SII was more effective and accurate in predicting the impact of T2DM on the immune system (47). Huaping Huang et al. found that SII can predict the postoperative survival rate of patients with cervical cancer, and it is more accurate and effective than other inflammatory factors (7). Hongmei Zhang et al. studied the relationship between leukocytes, centriocytes, lymphocytes, and diabetes, and the P values were all <0.001, and the final experiment found that the increase in leukocyte level was related to hyperglycemia. The research conclusion of Hongmei Zhang et al. is the same as ours (48). Saori Kashima et al., using the data from Yuport Medical Examination Center, also found that increased white blood cell count levels may increase the probability of diabetes, which is also consistent with our conclusion (49). Jin-Young Hwang et al. found that the prevalence of diabetes may increase with the increase of platelet count (50). This is consistent with our results. Yuqin Qian et al. found that platelet count may be related to diabetic peripheral neuropathy and is a potential risk marker (51). The SII selected in this study included platelet count, and the experimental results could truly reflect the burden of inflammation and accurately reflect the relationship between SII and diabetes.

The mechanisms underlying the positive association between inflammation and diabetes are unclear. Hitomi Usui Kataoka et al. suggested that endoplasmic reticulum stress may affect the pathogenesis of diabetes (52). Haichen Zhang et al. found epigenetic abnormalities in diabetic patients, which may provide information for target drug prediction (53). Mina Wang et al. summarized two causes of diabetes mellitus: impaired insulin action and impaired insulin secretion or a combination of both factors (54).

Our research has some advantages. The sample size is sufficient to be representative, and the years chosen are the most recent two data sets. We also adjusted for confounding factors to produce robust results. For example, previous studies have mentioned that dietary intake (55), physical activity (56) and protein intake (57) increase the prevalence of diabetes. As a result, we added protein intake and physical activity as factors to the fully adjusted model, which strengthened our findings. Our study does, however, have certain drawbacks. There is no causal association because it is a cross-sectional study, hence several prospective studies are required to explain the causative relationship. Confounding effects cannot be ruled out, even though we accounted for covariates to lessen their impact on the results.

## 5 Conclusion

More research is required to confirm our findings, which showed that SII levels are strongly related to diabetes.

## Data availability statement

The original contributions presented in the study are included in the article/supplementary material. Further inquiries can be directed to the corresponding author.

## Ethics statement

In 2003, the NHANES Institutional Review Board (IRB) changed its name to the NCHS Research Ethics Review Board (ERB). In 2018, the name was changed from NCHS Research Ethics Review Board to NCHS Ethics Review Board. The studies were conducted in accordance with the local legislation and institutional requirements. The participants provided their written informed consent to participate in this study.

## Author contributions

Conceptualization: YN and HZ. Methodology: YN and HZ. Software: YN and HZ. Validation: YN, HK, and HZ. Formal analysis: YN and HZ. Investigation: YN, HK, and HZ. Resources: YN, JW, HK, and HZ. Data curation: YN, HK, and HZ. Writing—original draft preparation: YN, and HZ. Writing—review and editing: HK. Visualization: YN. Supervision: YN. Project administration: YN, and HZ. Funding acquisition: YN, and JW. All authors have read and agreed to the published version of the manuscript.
